# Low-Frequency High-Magnitude Mechanical Strain of Articular Chondrocytes Activates p38 MAPK and Induces Phenotypic Changes Associated with Osteoarthritis and Pain

**DOI:** 10.3390/ijms150814427

**Published:** 2014-08-19

**Authors:** Derek H. Rosenzweig, Thomas M. Quinn, Lisbet Haglund

**Affiliations:** 1 The Orthopaedics Research Lab, Department of Surgery, McGill University, Montreal, QC H3G1A4, Canada; E-Mail: Derek.Rosenzweig@mail.mcgill.ca; 2 McGill Scoliosis & Spine Group, Department of Surgery, McGill University, Montreal, QC H3G1A4, Canada; 3 Department of Chemical Engineering, McGill University, Montreal, QC H3A2B2, Canada; E-Mail: thomas.quinn@rychiger.com

**Keywords:** articular cartilage, osteoarthritis, inflammation, pain, nerve growth factor, mechanical stretch, p38 MAPK

## Abstract

Osteoarthritis (OA) is a debilitating joint disorder resulting from an incompletely understood combination of mechanical, biological, and biochemical processes. OA is often accompanied by inflammation and pain, whereby cytokines associated with chronic OA can up-regulate expression of neurotrophic factors such as nerve growth factor (NGF). Several studies suggest a role for cytokines and NGF in OA pain, however the effects of changing mechanical properties in OA tissue on chondrocyte metabolism remain unclear. Here, we used high-extension silicone rubber membranes to examine if high mechanical strain (HMS) of primary articular chondrocytes increases inflammatory gene expression and promotes neurotrophic factor release. HMS cultured chondrocytes displayed up-regulated *NGF*, *TNFα* and *ADAMTS4* gene expression while decreasing TLR2 expression, as compared to static controls. HMS culture increased p38 MAPK activity compared to static controls. Conditioned medium from HMS dynamic cultures, but not static cultures, induced significant neurite sprouting in PC12 cells. The increased neurite sprouting was accompanied by consistent increases in PC12 cell death. Low-frequency high-magnitude mechanical strain of primary articular chondrocytes *in vitro* drives factor secretion associated with degenerative joint disease and joint pain. This study provides evidence for a direct link between cellular strain, secretory factors, neo-innervation, and pain in OA pathology.

## 1. Introduction

Articular cartilage functions in load bearing and smooth gliding motion of synovial joints. This function is directly attributed to the composition of the extracellular matrix (ECM), mainly comprised of collagen type II and proteogylcans [1,2]. A sparse population of chondrocytes within the ECM is responsible for both matrix synthesis and degradation. Articular cartilage is an avascular tissue with limited self-repair properties, and joint overload can cause a catabolic shift in chondrocyte phenotype by increasing expression of matrix metalloproteinases (MMPs) and aggrecanases (a disintegrin and metalloproteinase with thrombospondin motifs—ADAMTSs) initiating osteoarthritis (OA) [1,3]. Studies have suggested a link between OA, inflammation, neurotrophic factors and chronic pain, yet the mechanisms linking inflammatory pain with OA progression remain unclear [4].

Toll-like receptors (TLRs) are part of the superfamily of interleukin-1 (IL-1) receptors. TLRs detect outer pathogen components such as peptidoglycan from Gram-positive bacteria and lipopolysaccaride from Gram-negative bacteria [5] as well as cartilage matrix fragments [6], initiating an innate immune response. TLR activation often leads to cytokine release which contributes to inflammatory responses [7]. Additionally, mechanical strain can promote increased TLR expression in chondrocytes [8]. Since increased mechanical strains in OA cartilage leads to protease up-regulation [3], probable increases in matrix fragments may further promote TLR signalling, cytokine release and inflammatory responses. Mechanical strain may also affect cell homeostasis through mitogen activated protein kinases (MAPK) [9]. MAPKs consist of extracellular related kinase (ERK), p38 MAPK and c-JUN *N*-terminal kinase (JNK). We have recently shown that p38 signaling is involved in chondrocyte dedifferentiation [10], a process thought to be linked to disease progression. Effects of high magnitude mechanical strain on chondrocyte TLR expression, MAPK activity and inflammatory cytokine production have not been established.

We have developed a novel culture technique that facilitates more continuous growth of cells while limiting effects of contact inhibition and reducing the necessity for passaging [11,12]. Additionally, by using flexible silicone rubber dishes this culture device can apply low-frequency cyclic strain to modulate mesenchymal stem cell and C2C12 myoblast cell differentiation [13,14]. We also demonstrated that this device can apply high-magnitude near-injurious strain [15,16] to intervertebral disc (IVD) cells [17] resulting in increased NGF, TNF and inflammatory factor expression which may be related to low back pain. It is generally accepted that OA progression causes lower tissue compressive properties, resulting in higher tensile strains to resident cells—a process that can directly affect cell phenotype, metabolism and viability. Here, we use our dynamic culture device to apply high-magnitude stretch to primary chondrocytes at very low frequencies so as to avoid inducing cell detachment and death. We hypothesize that low-frequency high mechanical cyclical strain (20% at 0.0001 Hz) of primary articular chondrocytes can promote secretion of inflammatory and nociceptive factors associated with OA and joint pain.

## 2. Results

### 2.1. Low-Frequency Dynamic Culture Modulates Inflammatory and Matrix Remodelling Gene Expression

**Figure 1:**
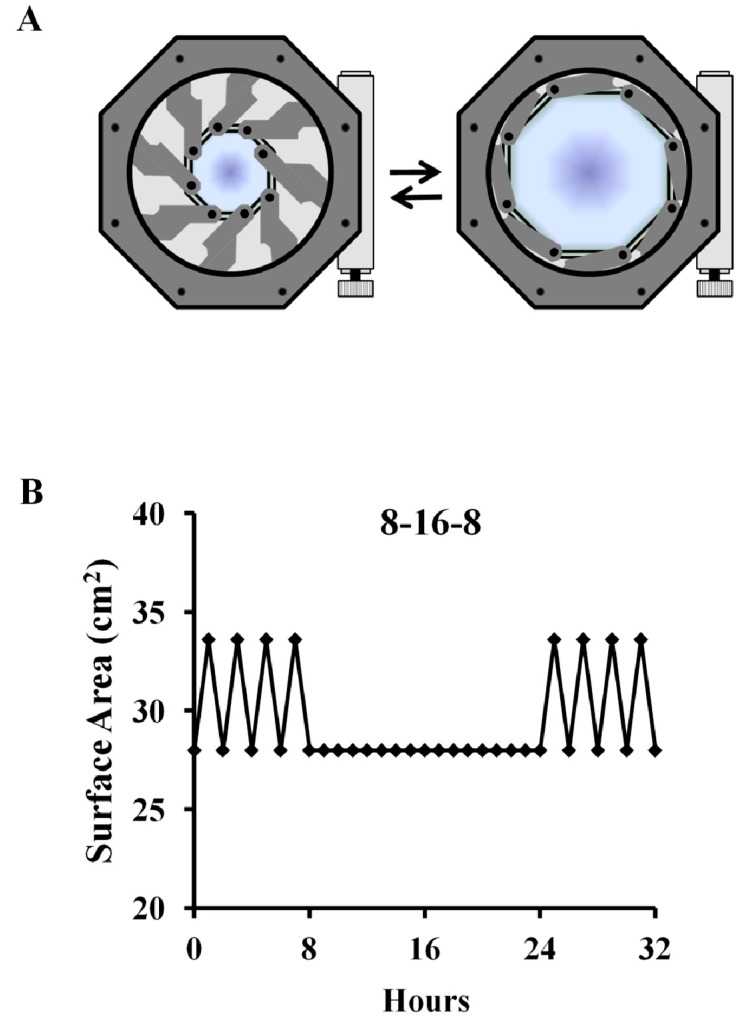
Cell stretching device and stretch protocol. Schematic representation of mechanical stretching device (**A**) used to apply low-frequency high-magnitude strains; (**B**) Graphical representation of the 8–16–8 and stretch protocol applied to primary chondrocytes.

**Figure 2:**
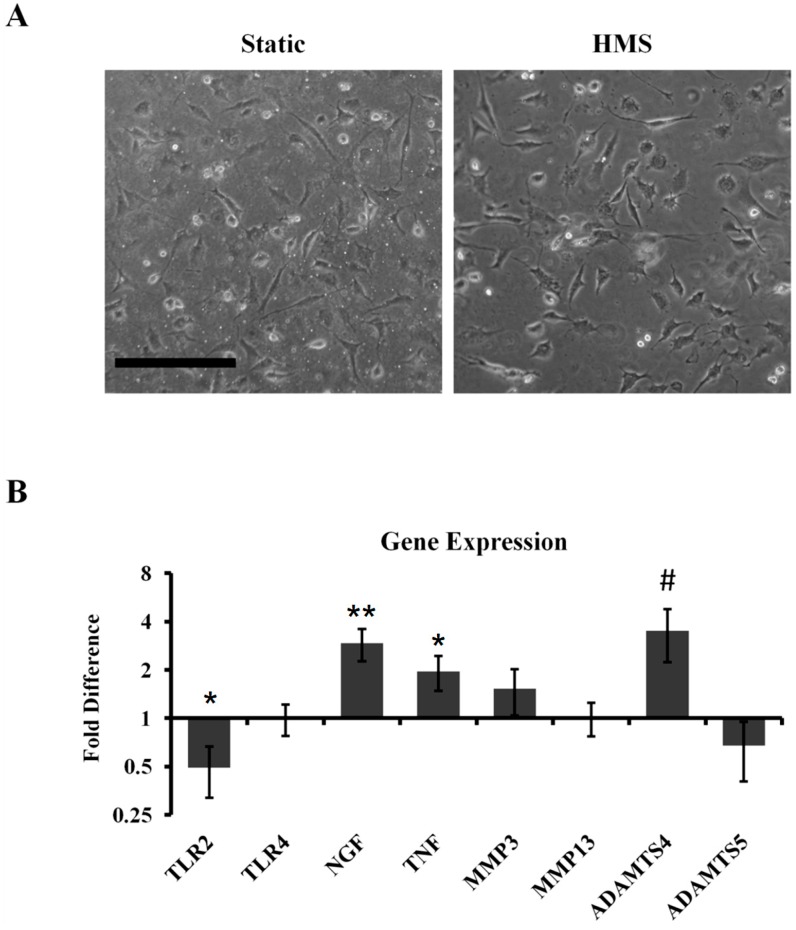
Application of the 8–16–8 stretch protocol to primary articular chondrocytes. (**A**) Representative morphological images of static and HMS cultured primary articular chondrocytes. Scale bar: 200 μm; (**B**) Gene expression analysis immediately after stretch protocol ended. Error bars: ±SEM, *n* = 6 (*TLR2*, *TLR4*, *NGF* and *TNF*); *n* = 3 (*MMP3*, *MMP13*, *ADAMTS4* and *ADAMTS5*). Student’s *t*-test. * indicates *p* < 0.05; ** indicates *p* < 0.01; # indicates *p* = 0.06.

We have previously established that cultures of primary cells, including chondrocytes, on chemically modified silicone surfaces alone do not influence physiological cell homeostasis [11,12,13,14,18]. High mechanical strain (HMS—20% stretch at 0.0001 Hz) was applied to primary chondorcytes for 8 h, followed by 16 h of rest and an additional 8 h of cyclic stretch (8–16–8, Figure 1). This was compared to cells cultured on static silicone surfaces, which were not subject to HMS. Cyclical dynamic HMS strain applied to the primary chondrocytes did not cause any dramatic effects to gross cell morphology (Figure 2A). Gene expression analysis revealed significantly increased levels of the neurotrophic and inflammatory factors *NGF* (2.93 ± 0.67 fold; *p* = 0.008) and *TNFα* (1.96 ± 0.48 fold; *p* = 0.034) compared to static cultured controls (Figure 2B). There was a strong trend for increased *ADAMTS4* expression (3.51 ± 1.27 fold; *p* = 0.06). *TLR2* expression was significantly decreased 2.03 ± 0.17 fold (*p* = 0.0071). No significant changes were detected for *TLR4*, *MMP3*, *MMP13* or *ADAMTS5* expression in dynamic *versus* static cultured chondrocytes (Figure 2B).

### 2.2. Low-Frequency Dynamic Culture of Primary Chondroctyes Increases p38 Activity

**Figure 3:**
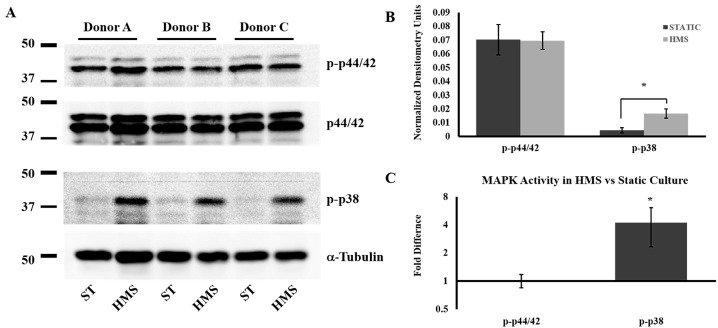
Western blot analysis. (**A**) Images acquired from immunoblots probing cell lysates from 3 donor animals (Donor A–C) probing for phosphorylated p44/42 (ERK), total p44/42 (ERK), phosphorylated p38, and for α-tubulin as a loading control; (**B**) Densitometry analysis of pERK normalized to total ERK and p-p38 normalized toα-tubulin; (**C**) Mean fold-difference in high mechanical strain (HMS) induced p-ERK and p-p38 activity *versus* static culture chondrocyte controls. Error bars: ±SEM, *n* = 3. Student’s *t*-test. * indicates *p* < 0.05. ST, static; HMS, high mechanical strain.

Since alterations in MAPK signaling are associated with chondrocyte dedifferentiation and HMS increased expression of genes associated with degenerative joint disease, we analyzed p44/42 (ERK) and p38 activity in HMS and static cultures. Western blot analysis showed no changes in phospho-ERK levels, yet revealed a consistent increase in phospho-p38 in the HMS culture cells (Figure 3A). Densitometry analysis of normalized MAPK activity (Figure 3B) from three individual experiments revealed a significant (4.22 ± 1.90)-fold increase in phospho-p38 in HMS cultured cells compared to static cultured cells (*p* = 0.0241) (Figure 3B). These data strongly indicate increased p38 activity in chondrocytes subjected to low-frequency high-magnitude strain.

### 2.3. Low-Frequency Dynamic Culture Drives Chondrocyte Secretion of Neurotrophic Factors

**Figure 4:**
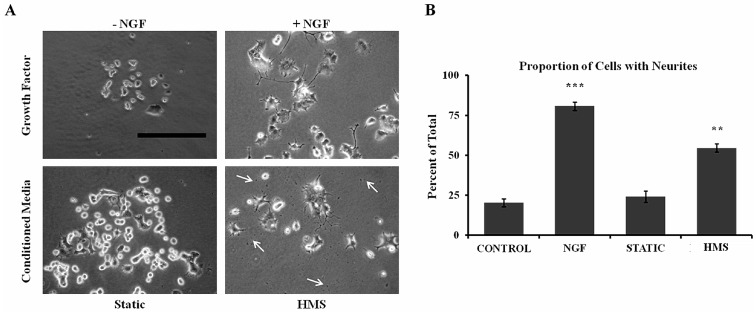
Conditioned media from HMS-cultured chondrocytes promotes neurite outgrowth in PC12 cells. (**A**) Representative phase images showing conditioned media from static and HMS cultured chondrocytes applied to PC12 cells and neurite outgrowth was observed and compared to vehicle (−NGF) and NGF treated controls. White arrows in phase images indicate noticeable cell debris on dish bottoms. Scale bar: 200 μm; (**B**) Quantification of neurite outgrowth. Error bars: ±SEM, *n* = 6, Student’s *t*-test. ** indicates *p* < 0.01; *** indicates *p* < 0.001. All samples were compared to −NGF controls.

The PC12 cell line is derived from rat pheochromocytoma, responds to NGF by sprouting axon-like neurites, and has been used extensively to study neuronal differentiation. To assess potential secretion of neurotrophic factors by HMS cultured chondrocytes, collected conditioned media from HMS and static cultures were applied to PC12 cells. After 4 days in culture, PC12 cells exposed to both vehicle or static culture conditioned media did not appear to have many neurites (Figure 4A). PC12 cells treated with both 50 ng/mL of NGF and HMS culture-conditioned media displayed a multitude of cells sprouting axon-like neurites (Figure 4A). Vehicle treated controls had a proportion of 20.38% ± 2.48% cells with neurites. NGF-treated PC12 cells displayed a significantly greater proportion of neurite containing cells (80.8% ± 2.62%, *p* = 0.00061). HMS conditioned media-treated cells also displayed a significantly higher proportion of neurite containing cells (54.72% ± 2.46%, *p* = 0.0079) as compared to vehicle controls. Static conditioned media-treated cells did not show any statistical difference in the proportion of neurite containing cells (24.19% ± 3.61%, *p* = 0.301) compared to controls (Figure 4B).

### 2.4. Low-Frequency Dynamic Chondrocyte Culture Drives Secretion of Cell Death-Promoting Factors

**Figure 5:**
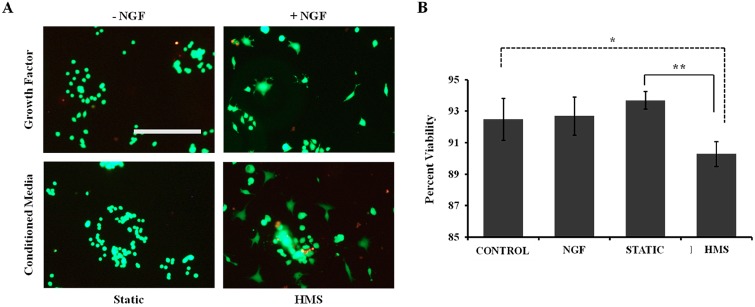
Conditioned media from HMS-cultured chondrocytes causes increased PC12 cell death. (**A**) Representative Live/Dead images showing conditioned media from static and HMS cultured chondrocytes applied to PC12 cells; viability was assessed and compared to vehicle (−NGF) and NGF treated controls. Green cells (Calcein AM) represent live cells, and red cells (Ethidium homodimer) indicate dead cells. Scale bar: 200 μm; (**B**) Quantification of viability. Error bars: ±SEM, *n* = 6, Student’s *t*-test. * indicates *p* < 0.05; ** indicates *p* < 0.01. All samples were compared to −NGF controls.

In a parallel study on effects of low-frequency high-magnitude strain on human intervertebral disc cells, conditioned media from HMS intervertebral disc cell cultures caused significantly increased cell death when applied to PC12 cells [17]. Here, PC12 cells exposed to HMS chondrocyte cultured media consistently displayed traces of cell debris in culture dishes (Figure 4A, white arrows). Compared to vehicle treated control PC12 viability (93.2% ± 1.34%), Live/Dead assay revealed a consistently small, but significant reduction in viability (90.3% ± 0.79%, *p* = 0.016) when PC12 cells were exposed to the dynamic media (Figure 5). NGF treatment displayed 92.7% ± 1.21% viable cells. PC12 cells treated with static culture media displayed 93.7% ± 0.56% viable cells, which was also significantly higher than cells treated with the dynamic conditioned media (*p* = 0.0065) (Figure 5).

## 3. Discussion

Changing biomechanical properties within degenerating joints can influence and promote inflammatory and cytokine responses, increasing factors that have been associated with joint pain in OA. To elucidate the cellular and molecular effects of high mechanical strain, primary bovine chondrocytes were subjected to HMS culture in our unique dynamic culture device coupled to high-extension silicone rubber (HESR) culture dishes. Gene expression, MAPK activity, and conditioned media were analyzed revealing suppressed *TLR* gene expression and an up-regulation of *NGF*, *TNF*, *MMP* and *ADAMTS* gene expression. The altered gene expression was associated with increased p38 activity. Furthermore, conditioned media from dynamically cultured cells was able to induce significant neurite outgrowth in PC12 cells. The conditioned media, however, also caused an increase in cell death compared to static, NGF- and vehicle-treated controls. These data indicate that adverse mechanical strain can cause isolated chondrocytes to assume a more degenerate-like phenotype and secrete factors (NGF, TNF) which have been associated with cartilage degeneration and pain.

Several clinical and animal studies have shown that excessive mechanical loading can disrupt matrix composition [9], alter cell metabolism and gene expression [19] and change overall mechanical properties in articular cartilage [20,21]. It is quite possible that such changing biomechanics within early degenerate tissue applies higher mechanical strains to drive inflammatory and catabolic gene expression *in vivo*. Several reports have suggested that increased TNFα and IL1β (among other inflammatory factors) are increased in synovial tissue of OA patients. Additionally, nociceptive agents NGF and brain-derived neurotrophic factor (BDNF) have also been observed in OA patients. In the functionally similar intervertebral disc tissue, we have observed these same factors in surgical samples from patients suffering chronic axial back pain [22]. Moreover, HMS applied to isolated healthy IVD cells induced NGF and TNF production, which stimulated PC12 cell neurite sprouting. Taken together, our data suggest that adverse mechanical stretch promotes the production of factors associated with tissue degeneration and pain.

TLR signalling has recently been implicated in osteoarthritis, cartilage degeneration and pain. TLR2 and TLR4 receptors are both expressed by osteoarthritic chondrocytes [23,24] as well as in synovial tissue of rheumatoid arthritis patients [25]. TLR signalling results in increased production of inflammatory factors associated with chronic arthritis pain [26,27]. We have reported that HMS strain applied to human intervertebral disc cells can increase *TLR2* and *TLR4* gene expression [17]. Interestingly, the exact same HMS strain applied to primary bovine articular chondrocytes had no effect on *TLR4* expression and significantly reduced *TLR2* transcript levels. This may be a unique difference between IVD cells and chondrocytes of the knee, or it may be due to species differences between bovine and human samples. Future experiments will evaluate the effects of HMS strain on isolated human articular chondrocytes.

In addition to probing for TLR receptor expression, the present study probed HMS cultured chondrocytes for gene markers often associated with cartilage degeneration and pain. TNFα has been strongly associated with both joint degeneration [28,29] and pain [30,31]. HMS caused a significant up-regulation of *TNFα* in primary chondrocytes, suggesting a biomechanical role for increased TNF in OA. HMS culture also caused significantly increased expression of the neurotrophic factor NGF. Increases in the active form of NGF have been directly linked with progression of degenerative joint [32] and degenerative disc disease [22,33], and anti-NGF therapies have had some success in pain alleviation [34]. HMS culture of primary chondrocytes also caused a trend for increased expression of the matrix metalloproteinase 3 (*MMP3*) and the aggrecanase *ADAMTS4*, both of which are classically involved in joint degeneration [35,36]. Combined with recent findings in HMS culture of IVD cells [17], our present findings also suggest a mechanical role for increased expression of factors involved in a degenerate phenotype in isolated articular chondrocytes.

The MAP kinases, among other signal transduction proteins, can transmit cellular stress signals to alter cell phenotype and behavior. Modulation of ERK and p38 activities has been shown in models of both mechanically strained cartilage and early OA [37,38,39]. When an adverse cyclical strain of 20% was applied to primary chondrocytes in this study, increased p38 activity was observed possibly due to mechanical activation of integrins [40]. This data strongly correlates with our previous findings in an acute mechanical injury model in osteochondral explants [9], suggesting that increased p38 activity may contribute to phenotypic changes of chondrocytes under high mechanical strains. Interestingly, p38 activity is also involved in chondrocyte dedifferentiation [10] a cellular process that has been linked to degenerative disease. Similar to OA progression, both p38 and ERK signalling have been implicated in intervertebral disc degeneration and inflammation [41]. Here, HMS culture of primary chondrocytes showed significantly increased p38 activity, which may indicate a phenotypic switch of the cells. Further studies on the exact mechanism through which mechanical strain-induces p38 activity and drives these phenotypic changes in chondrocytes may yield important insights for novel therapeutics to combat joint degeneration and pain.

Healthy articular cartilage tissue is avascular and aneural. Degradation of the tissue and remodeling of the underlying bone during disease progression can lead to neo-vascularization and nerve fiber in-growth [42] to the tissue and surrounding synovium. This sets the stage for inflammation and pain in OA. We investigated the ability of HMS cultured chondrocytes to produce factors which can stimulate innervation. PC12 cells have been used in a variety of studies for neuronal differentiation and neurite outgrowth including HMS cultures [17]. Here, HMS conditioned media was able to drive significant neurite outgrowth in PC12 cells while the static culture conditioned media did not. This data suggests that HMS culture not only causes increased *NGF* gene expression but also produces enough protein levels of NGF to stimulate PC12 cell differentiation towards a neuronal phenotype. The HMS conditioned media also contained factors which promoted a slight, but consistent increase in PC12 cell death. This may be due to the observed increase in factors such TNFα, which is known to induce PC12 cell death [43]. HMS culture has also been shown to cause increased inflammatory factor secretion in human IVD cells, which also may play a role in the observed cell death [17]. Taken together, these *in vitro* findings indicate that changes in mechanical properties in OA cartilage can drive articular chondrocytes to produce factors, including NGF, which can potentially stimulate neuronal ingrowth, cell death and pain *in vivo*.

Our culture device was initially used to culture large populations of phenotypically enhanced primary chondrocytes for tissue engineering applications [11]. Another advantage of this device is the application of high magnitude cyclical strains at very low frequencies, thereby avoiding induced cell death. Application of mechanical strains over 20% combined with frequencies above 0.5 Hz have been associated with apoptosis and cell death [44]. A recent report showed that cyclic mechanical compression of cartilage explants causes secretion of NGF [45], however the frequency of cyclic strain was 0.5 Hz (1 MPa) while no viability analysis was performed and no functional assessment was made with the collected conditioned media. Also, explants used in that study were dissected from the murine rib cage. That type of mechanical strain used may have caused trauma and cell death. We have successfully used low frequency cyclical strain to modulate mesenchymal stem cell [14] and C2C12 myoblast cell line [13] differentiation and lineage specification. The exact HMS strain protocol used in this study was also used to drive similar effects of increased NGF and other factors described here in human nucleus pulposus and annulus fibrosus cells isolated from intervertebral discs [17]. Interestingly, HMS culture of IVD cells promotes release of factors which can drive significant PC12 cell death, whereas conditioned media from HMS culture of chondroctyes in this study did not produce this result in PC12 cells. This demarcation in stretch-bearing capacities between disc cells and chondrocytes suggests that cartilage may resist HMS more than IVD tissue. Future studies will focus on mechanisms by which HMS exerts its effects on chondrocyte phenotype. Also, more in-depth studies will examine high mechanical strain to cells cultured in 3D matricies which may yield important insights to the biomechanical role in OA disease progression, inflammatory factor secretion, tissue innervation and pain.

## 4. Experimental Section

### 4.1. Chondrocyte Isolation

Primary bovine chondrocytes were isolated as described previously [11,12]. Briefly, knee joints from freshly slaughtered skeletally mature cows were obtained from a local slaughterhouse. Approximately 5 g of tissue was washed in sterile phosphate buffered saline (PBS) supplemented with antibiotics and cut into 1–2 mm pieces. The tissue was digested overnight in a T-75 flask containing 30 mL of chondrocyte growth medium (high-glucose DMEM; 0.1 mM Nonessential Amino Acids; 10 mM HEPES; 1 mM sodium pyruvate; 10% fetal bovine serum; and 1% penicillin–streptomycin–glycine solution) supplemented with 1.5 mg/mL collagenase type II (Invitrogen/Gibco, Burlington, ON, Canada). The digest was passed through a 100 µm filter (BD Biosciences, Mississauga, ON, Canada) and centrifuged at 300× *g* for 5 min. Pelleted chondrocytes were washed with sterile PBS and centrifuged again at 300× *g* for 5 min. Cells were resuspended in 10 mL of chondrocyte growth medium and counted.

### 4.2. Static Silicone and High Mechanical Dynamic Strain

All silicone surfaces were salinized and coated with collagen type I to promote cell adhesion exactly as described previously [11,12,13,14,17,46]. Primary chondrocytes (200,000 cells) were seeded on high-extension silicone rubber (HESR) dishes (28 cm^2^ initial surface area) and static control 60 mm polystyrene culture dishes coated with approximately 500 µm of silicone rubber termed static silicone (Static Culture) (Factor II—surface area 28 cm^2^). Both static and HESR dishes were cultured for 48 h with cells in their respective medium prior to starting stretch protocols, as described above supplemented with 10% FBS. All silicone rubber culture surfaces were chemically modified and coated with rat tail collagen type I (50 µg/mL; Sigma-Aldrich, Oakville, ON, Canada) to promote cell adhesion as previously described [12,14,18,46,47].

After 48 h of seeding cells, the growth surface was rinsed twice with sterile PBS and respective culture media were replaced with serum-free medium supplemented with 1× ITS solution (Insulin–Transferrin–Selenium, Gibco Invitrogen, Burlington, ON, Canada). With static culture (surface area 28 cm^2^) as control, HESR cultures were subjected to 8 h high mechanical strain (HMS—20% cyclical strain at frequency of 0.0001 Hz) followed by 16 h of intervening rest and another 8 h of cyclic dynamic strain using an iris-like stretching device coupled to the HESR dishes (Figure 1A,B). After the “8–16–8” stretch protocol, the resulting conditioned media was collected immediately after the final stretch period ended. At the same time, cells were immediately lysed directly in TRIzol or cell lysis buffer for RNA and protein isolation.

### 4.3. RNA Isolation and Real Time qPCR

Cells were lysed directly on culture surfaces using 1ml TRIzol (Invitrogen) reagent and collected in nuclease-free tubes. Following RNA extraction according to the manufacturer’s protocol, 500 ng of total RNA was subject to cDNA synthesis using the qScript cDNA synthesis kit following the manufacturer’s instructions (Quanta Biosciences, Gaithersburg, MD, USA). RNA concentrations and purity were determined by measuring A_260_ by calculating the A_260_/A_280_ ratio using a nano-drop method (Infinite M200 Pro, TECAN, Mannedorf, Switzerland). Standard recommended PCR protocols were performed (50 °C for 2 min, 94 °C for 10 min, 95 °C for 30 s, 60 °C for 1 min, with steps 3 and 4 repeated for 40 cycles) using the ABI STEP ONE Real-Time PCR System (Applied Biosystems, Carlsbad, CA, USA). The average cycle count for each target gene was normalized to mammalian 18 s to give the average delta count (∆*C*_t_) using RQ SDS manager software (Applied Biosystems). Commercially available Taqman array primers were used, and genes analyzed were *TLR-2* (Cat No. Bt03223212_m1), *TLR-4* (Cat No. Bt03251670_m1), *NGF* (Cat No. Bt03817604_s1) and TNF (Cat No. Bt03259154_m1), *MMP3* (Cat No. Bt04259490_m1), *MMP13* (Cat No. Bt03214050_m1), *ADAMTS4* (Cat No. Bt03224693_m1), *ADAMTS5* (Cat No. Bt04230785_m1), and with *18S* (Cat No. Hs99999901_s1) as the endogenous control. Gene expression was calculated using the ∆∆*C*_t_ method [48]. For *TLR2*, *TLR4*, *NGF* and *TNF* assessment, *n* = 6. For *MMP3*, *MMP13*, *ADAMTS4* and *ADAMTS5* assessment, *n* = 3.

### 4.4. Induction of Neurite Outgrowth Using Conditioned Media

For all experiments, 2 × 10^5^ PC12 cells/well (passage 2) (ATCC, Manassas, VA, USA) were seeded on 6-well culture dishes coated with 50 µg/mL of collagen type I and 0.1% Poly-l-Lysine (70–150 kD; Sigma). Cells were allowed to attach to culture surfaces for 24 h in RPMI 1640 medium supplemented with 1% Antibiotic-Antimycotic solution, 5% FBS and 10% Horse serum (all from Gibco/Invitrogen). After 24 h, control wells were changed to 0.1% serum chondrocyte growth medium supplemented with 50 ng/mL of recombinant human β-NGF (nerve growth factor—Bioshop, Burlington, ON, Canada) or sterile water vehicle. Remaining wells received 1.5 mL of conditioned media collected from experiments above (Static and HMS-cultured chondrocytes). Neurite outgrowth was monitored for 4 days. Three random phase images per sample were taken from each individual experiment (*n* = 6) and the proportion of cells with neurites was quantified. Phase images were captured using a Zeiss Axiovert 40C microscope equipped with a Canon Powershot A640 digital camera attached to a Zeiss MC80DX 1.0× tube adapter. Cell viability was assessed by Live/Dead^®^ (Invitrogen) assay according to the vendor’s instruction and total dead (red) and live (green) cells were counted from 3 random positions from 6 independent experiments (*i.e.*, 18 images total for static-cultured conditioned media treated PC12 cells; 18 images total for HMS-cultured conditioned media treated PC12 cells, *etc.*). The percentage of live cells was calculated and averaged. An Olympus IX81 inverted fluorescence microscope was used, and all images were captured using a 10× objective with MAG Biosystems Software 7.5 (Photometrics, Tucson, AZ, USA).

### 4.5. Western Blot

Protein concentration was determined by Bradford assay using a TECAN Infinite M200 Pro for static and HMS chondrocytes lysates (20 mM Tris (pH 7.4), 150 mM NaCl, 1 mM EDTA, 0.5% Triton X-100, 1 mM β-glycerophosphate, supplemented with complete EDTA-free protease inhibitor cocktail). Twenty micrograms total protein of each sample was subjected to 12% SDS-PAGE gel electrophoresis and then transferred to nitrocellulose membranes. Membranes were blocked in 5% BSA for 45 min and probed with rabbit polyclonal antibodies against p-p44/42 (p-ERK) (1:1000; Cell Signaling #4370, Danvers, MA, USA), p44/42 (total ERK-1:1000; Cell Signaling #4695) and p-p38 (1:2000; Cell Signaling #9211), and mouse monoclonal antibodies against α-tubulin (1:1000; Abcam Ab7291). Membranes were washed three times in TBST followed by incubation with either anti-rabbit HRP-conjugated secondary antibody (1:5000, Santa Cruz Biotechnology, Santa Cruz, CA, USA) or anti-mouse HRP-conjugated secondary antibody (1:3000, Santa Cruz Biotechnology). Membranes were then washed three times in TBST for 10 min, and developed using Western Lightning Plus-ECL (Perkin Elmer, Waltham, MA, USA) and an Image Quant LAS 4000 (GE Healthcare Bio-Sciences, Baie d’Urfe, QC, Canada) was used to capture images and perform densitometry analysis within the linear exposure range. Phospho-ERK bands were normalized to total ERK bands, and phospho-p38 bands were normalized to α-tubulin.

### 4.6. Statistical Analysis

All values are represented as means ± standard error of the mean of three to six independent experiments with at least three different animal donors for each experiment. All comparisons were made between the two experimental groups, HMS and static cultures. No multiple comparisons were made. Differences between HMS and static groups were assessed using a paired two-tailed Student’s *t*-test with *post hoc* Bonferroni correction. Differences were considered significant for *p* < 0.05.

## 5. Conclusions

Changing mechanical properties within degenerate cartilage tissue will gradually apply higher strain to resident chondrocytes which is thought to cause a more catabolic environment, thereby contributing to OA progression. High mechanical strain applied to primary chondrocytes in this study caused p38 MAPK activity and up-regulated expression of inflammatory factors known to drive catabolic processes. Conditioned media from HMS cultures caused significant neurite sprouting in PC12 cells indicating sufficient factor secretion to drive neurogenesis. Taken together, these data suggest that high mechanical strain can promote an inflammatory and degenerate phenotype in chondrocytes *in vitro*. Since OA progression is a slow process typically taking years to develop, this low-frequency high-magnitude strain may therefore be related to OA matrix degradation, inflammation and pain *in vivo*.

## References

[1] Anderson D.D., Chubinskaya S., Guilak F., Martin J.A., Oegema T.R., Olson S.A., Buckwalter J.A. (2011). Post-traumatic osteoarthritis: Improved understanding and opportunities for early intervention. J. Orthop. Res..

[2] Bhosale A.M., Richardson J.B. (2008). Articular cartilage: Structure, injuries and review of management. Br. Med. Bull..

[3] Natoli R.M., Athanasiou K.A. (2009). Traumatic loading of articular cartilage: Mechanical and biological responses and post-injury treatment. Biorheology.

[4] Lee A.S., Ellman M.B., Yan D., Kroin J.S., Cole B.J., van Wijnen A.J., Im H.J. (2013). A current review of molecular mechanisms regarding osteoarthritis and pain. Gene.

[5] Akira S., Takeda K. (2004). Toll-like receptor signalling. Nat. Rev. Immunol..

[6] Haglund L., Bernier S.M., Onnerfjord P., Recklies A.D. (2008). Proteomic analysis of the LPS-induced stress response in rat chondrocytes reveals induction of innate immune response components in articular cartilage. Matrix Biol..

[7] Sillat T., Barreto G., Clarijs P., Soininen A., Ainola M., Pajarinen J., Korhonen M., Konttinen Y.T., Sakalyte R., Hukkanen M. (2013). Toll-like receptors in human chondrocytes and osteoarthritic cartilage. Acta Orthop..

[8] Wang P., Zhu F., Tong Z., Konstantopoulos K. (2011). Response of chondrocytes to shear stress: Antagonistic effects of the binding partners Toll-like receptor 4 and caveolin-1. FASEB J..

[9] Rosenzweig D.H., Djap M.J., Ou S.J., Quinn T.M. (2012). Mechanical injury of bovine cartilage explants induces depth-dependent, transient changes in MAP kinase activity associated with apoptosis. Osteoarthr. Cartil..

[10] Rosenzweig D.H., Ou S.J., Quinn T.M. (2013). P38 mitogen-activated protein kinase promotes dedifferentiation of primary articular chondrocytes in monolayer culture. J. Cell. Mol. Med..

[11] Rosenzweig D.H., Matmati M., Khayat G., Chaudhry S., Hinz B., Quinn T.M. (2012). Culture of primary bovine chondrocytes on a continuously expanding surface inhibits dedifferentiation. Tissue Eng. A.

[12] Rosenzweig D.H., Solar-Cafaggi S., Quinn T.M. (2012). Functionalization of dynamic culture surfaces with a cartilage extracellular matrix extract enhances chondrocyte phenotype against dedifferentiation. Acta Biomater..

[13] Khayat G., Rosenzweig D.H., Khavandgar Z., Li J., Murshed M., Quinn T.M. (2013). Low-frequency mechanical stimulation modulates osteogenic differentiation of C2C12 Cells. ISRN Stem Cells.

[14] Khayat G., Rosenzweig D.H., Quinn T.M. (2012). Low frequency mechanical stimulation inhibits adipogenic differentiation of C3H10T1/2 mesenchymal stem cells. Differentiation.

[15] Stokes I.A. (1987). Surface strain on human intervertebral discs. J. Orthop. Res..

[16] Ianuzzi A., Pickar J.G., Khalsa P.S. (2010). Validation of the cat as a model for the human lumbar spine during simulated high-velocity, low-amplitude spinal manipulation. J. Biomech. Eng..

[17] Gawri R., Rosenzweig D.H., Krock E., Ouellet J.A., Stone L.S., Quinn T.M., Haglund L. (2014). High mechanical strain of primary intervertebral disc cells promotes secretion of inflammatory factors associated with disc degeneration and pain. Arthritis Res. Ther..

[18] Majd H., Wipff P.J., Buscemi L., Bueno M., Vonwil D., Quinn T.M., Hinz B. (2009). A novel method of dynamic culture surface expansion improves mesenchymal stem cell proliferation and phenotype. Stem Cells.

[19] Fitzgerald J.B., Jin M., Grodzinsky A.J. (2006). Shear and compression differentially regulate clusters of functionally related temporal transcription patterns in cartilage tissue. J. Biol. Chem..

[20] Quinn T.M., Allen R.G., Schalet B.J., Perumbuli P., Hunziker E.B. (2001). Matrix and cell injury due to sub-impact loading of adult bovine articular cartilage explants: Effects of strain rate and peak stress. J. Orthop. Res..

[21] Buckwalter J.A., Mankin H.J., Grodzinsky A.J. (2005). Articular cartilage and osteoarthritis. Instr. Course Lect..

[22] Krock E., Rosenzweig D.H., Chabot-Doret A.J., Jarzem P., Weber M.H., Ouellet J.A., Stone L.S., Haglund A. (2014). Painful degenerating intervertebral discs up-regulate neurite sprouting and CGRP through nociceptive factors. J. Cell. Mol. Med..

[23] Barreto G., Sillat T., Soininen A., Ylinen P., Salem A., Konttinen Y.T., Al-Samadi A., Nordstrom D.C. (2013). Do changing toll-like receptor profiles in different layers and grades of osteoarthritis cartilage reflect disease severity?. J. Rheumatol..

[24] Liu-Bryan R., Terkeltaub R. (2010). Chondrocyte innate immune myeloid differentiation factor 88-dependent signaling drives procatabolic effects of the endogenous Toll-like receptor 2/Toll-like receptor 4 ligands low molecular weight hyaluronan and high mobility group box chromosomal protein 1 in mice. Arthritis Rheumatol..

[25] Proost P., Vynckier A.K., Mahieu F., Put W., Grillet B., Struyf S., Wuyts A., Opdenakker G., van Damme J. (2003). Microbial Toll-like receptor ligands differentially regulate CXCL10/IP-10 expression in fibroblasts and mononuclear leukocytes in synergy with IFN-γ and provide a mechanism for enhanced synovial chemokine levels in septic arthritis. Eur. J. Immunol..

[26] Guerrero A.T., Cunha T.M., Verri W.A., Gazzinelli R.T., Teixeira M.M., Cunha F.Q., Ferreira S.H. (2012). Toll-like receptor 2/MyD88 signaling mediates zymosan-induced joint hypernociception in mice: Participation of TNF-α, IL-1β and CXCL1/KC. Eur. J. Pharmacol..

[27] Christianson C.A., Dumlao D.S., Stokes J.A., Dennis E.A., Svensson C.I., Corr M., Yaksh T.L. (2011). Spinal TLR4 mediates the transition to a persistent mechanical hypersensitivity after the resolution of inflammation in serum-transferred arthritis. Pain.

[28] Westacott C.I., Barakat A.F., Wood L., Perry M.J., Neison P., Bisbinas I., Armstrong L., Millar A.B., Elson C.J. (2000). Tumor necrosis factor alpha can contribute to focal loss of cartilage in osteoarthritis. Osteoarthr. Cartil..

[29] Stannus O., Jones G., Cicuttini F., Parameswaran V., Quinn S., Burgess J., Ding C. (2010). Circulating levels of IL-6 and TNF-α are associated with knee radiographic osteoarthritis and knee cartilage loss in older adults. Osteoarthr. Cartil..

[30] Hess A., Axmann R., Rech J., Finzel S., Heindl C., Kreitz S., Sergeeva M., Saake M., Garcia M., Kollias G. (2011). Blockade of TNF-α rapidly inhibits pain responses in the central nervous system. Proc. Natl. Acad. Sci. USA.

[31] Kochukov M.Y., McNearney T.A., Yin H., Zhang L., Ma F., Ponomareva L., Abshire S., Westlund K.N. (2009). Tumor necrosis factor-alpha (TNF-α) enhances functional thermal and chemical responses of TRP cation channels in human synoviocytes. Mol. Pain.

[32] Raychaudhuri S.P., Raychaudhuri S.K., Atkuri K.R., Herzenberg L.A. (2011). Nerve growth factor: A key local regulator in the pathogenesis of inflammatory arthritis. Arthritis Rheumatol..

[33] Purmessur D., Freemont A.J., Hoyland J.A. (2008). Expression and regulation of neurotrophins in the nondegenerate and degenerate human intervertebral disc. Arthritis Res. Ther..

[34] Garber K. (2011). Fate of novel painkiller mAbs hangs in balance. Nat. Biotechnol..

[35] Kubota E., Imamura H., Kubota T., Shibata T., Murakami K. (1997). Interleukin 1β and stromelysin (MMP3) activity of synovial fluid as possible markers of osteoarthritis in the temporomandibular joint. J. Oral Maxillofac. Surg..

[36] Tortorella M.D., Malfait A.M., Deccico C., Arner E. (2001). The role of ADAM-TS4 (aggrecanase-1) and ADAM-TS5 (aggrecanase-2) in a model of cartilage degradation. Osteoarthr. Cartil..

[37] Fanning P.J., Emkey G., Smith R.J., Grodzinsky A.J., Szasz N., Trippel S.B. (2003). Mechanical regulation of mitogen-activated protein kinase signaling in articular cartilage. J. Biol. Chem..

[38] Sondergaard B.C., Schultz N., Madsen S.H., Bay-Jensen A.C., Kassem M., Karsdal M.A. (2010). MAPKs are essential upstream signaling pathways in proteolytic cartilage degradation—Divergence in pathways leading to aggrecanase and MMP-mediated articular cartilage degradation. Osteoarthr. Cartil..

[39] Takebe K., Nishiyama T., Hayashi S., Hashimoto S., Fujishiro T., Kanzaki N., Kawakita K., Iwasa K., Kuroda R., Kurosaka M. (2011). Regulation of p38 MAPK phosphorylation inhibits chondrocyte apoptosis in response to heat stress or mechanical stress. Int. J. Mol. Med..

[40] Aikawa R., Nagai T., Kudoh S., Zou Y., Tanaka M., Tamura M., Akazawa H., Takano H., Nagai R., Komuro I. (2002). Integrins play a critical role in mechanical stress-induced p38 MAPK activation. Hypertension.

[41] Wuertz K., Haglund L. (2013). Inflammatory mediators in intervertebral disk degeneration and discogenic pain. Global Spine J..

[42] Suri S., Gill S.E., Massena de Camin S., Wilson D., McWilliams D.F., Walsh D.A. (2007). Neurovascular invasion at the osteochondral junction and in osteophytes in osteoarthritis. Ann. Rheum. Dis..

[43] Koski C.L., Hila S., Hoffman G.E. (2004). Regulation of cytokine-induced neuron death by ovarian hormones: Involvement of antiapoptotic protein expression and c-JUN *N*-terminal kinase-mediated proapoptotic signaling. Endocrinology.

[44] Zhang Y.H., Zhao C.Q., Jiang L.S., Dai L.Y. (2011). Cyclic stretch-induced apoptosis in rat annulus fibrosus cells is mediated in part by endoplasmic reticulum stress through nitric oxide production. Eur. Spine J..

[45] Pecchi E., Priam S., Gosset M., Pigenet A., Sudre L., Laiguillon M.C., Berenbaum F., Houard X. (2014). Induction of nerve growth factor expression and release by mechanical and inflammatory stimuli in chondrocytes: Possible involvement in osteoarthritis pain. Arthritis Res. Ther..

[46] Rosenzweig D.H., Chicatun F., Nazhat S.N., Quinn T.M. (2013). Cartilaginous constructs using primary chondrocytes from continuous expansion culture seeded in dense collagen gels. Acta Biomater..

[47] Wipff P.J., Majd H., Acharya C., Buscemi L., Meister J.J., Hinz B. (2009). The covalent attachment of adhesion molecules to silicone membranes for cell stretching applications. Biomaterials.

[48] Livak K.J., Schmittgen T.D. (2001). Analysis of relative gene expression data using real-time quantitative PCR and the 2^−∆∆*C*t^ method. Methods.

